# Impact of Epstein-Barr Virus on Peripheral T-Cell Lymphoma Not Otherwise Specified and Angioimmunoblastic T-Cell Lymphoma

**DOI:** 10.3389/fonc.2021.797028

**Published:** 2022-01-11

**Authors:** Tong-Yoon Kim, Gi-June Min, Young-Woo Jeon, Sung-Soo Park, Silvia Park, Seung-Hawn Shin, Seung-Ah Yahng, Jae-Ho Yoon, Sung-Eun Lee, Byung-Sik Cho, Ki-Seong Eom, Yoo-Jin Kim, Seok Lee, Hee-Je Kim, Chang-Ki Min, Jong-Wook Lee, Seok-Goo Cho

**Affiliations:** ^1^ Department of Hematology, Seoul St. Mary’s Hematology Hospital, College of Medicine, The Catholic University of Korea, Seoul, South Korea; ^2^ Department of Hematology, Yeouido St. Mary’s Hematology Hospital, College of Medicine, The Catholic University of Korea, Seoul, South Korea; ^3^ Department of Hematology, Eunpyeong St. Mary’s Hematology Hospital, College of Medicine, The Catholic University of Korea, Seoul, South Korea; ^4^ Department of Hematology, Incheon St. Mary’s Hospital, College of Medicine, The Catholic University of Korea, Seoul, South Korea

**Keywords:** Epstein-Barr virus, angioimmunoblastic T-cell lymphoma, peripheral T-cell lymphoma, T-cell lymphoma, transplantation

## Abstract

**Purpose:**

The significance of Epstein-Barr virus (EBV) infections for the prognosis of patients with peripheral T-cell lymphomas (PTCLs), specifically angioimmunoblastic T-cell lymphoma (AITL) and PTCL not otherwise specified (PTCL-NOS), remains unclear. The Epstein-Barr encoding region can be used to detect EBV in tissue sections by *in situ* hybridization (ISH) and by polymerase chain reaction (PCR) assays of peripheral blood samples from patients with PTCLs. This study compared the outcomes patients with AITL or PTCL-NOS for whom the presence of EBV infection was assessed by these two methods.

**Patients and Methods:**

This was a retrospective study of patients newly diagnosed with AITL or PTCL-NOS. All patients were selected from a single transplantation center. EBV-positive lymphomas were detected at the time of diagnosis in tissue sections by ISH or in the blood by PCR.

**Results:**

Out of a cohort of 140 patients with histologically confirmed AITL or PTCL-NOS, 105 were EBV-positive. The 3-year overall survival of patients with EBV-positive TCL was 43.3% compared to 68.6% in patients with EBV-negative TCL (p = .01). Patients who were treated with autologous or allogeneic hematopoietic stem cell transplantation (n = 28 and n = 11, respectively) or chemotherapy alone (n = 66) had 3-year survival rates of 67.0%, 62.3%, and 30.2%, respectively (p <.02). Patients with EBV-positive TCL had a better prognosis after treatment with hematopoietic stem cell transplantation compared to chemotherapy alone, but no difference was seen among patients with EBV-negative TCL.

**Conclusions:**

EBV infection was shown to negatively affect the clinical outcomes of patients with TCL. Stem cell transplantation has been found to be an effective treatment for EBV-associated lymphomas. Further investigations are warranted to determine the optimal treatment for these patients.

## Introduction

Peripheral T-cell lymphomas (PTCLs) are neoplasms derived from the post-thymic lymphocytes and account for approximately 15% of non-Hodgkin lymphoma in the Western hemisphere (1). Angioimmunoblastic T-cell lymphoma (AITL) and PTCL not otherwise specified (PTCL-NOS) are the second and third most common PTCLs in Asia and account for 24.7% and 20.8% of cases, respectively (2).

PTCL-NOS is diagnosed after excluding other subtypes and consists of both histologically and immunohistochemically heterogeneous tumors (3). Epstein-Barr virus (EBV) is detected in approximately 74-91% of all AITL cases and about 21% of PTCL-NOS cases (4, 5).

The effect of EBV on PTCLs has long been debated. According to the literature, some studies have reported a worse prognosis in patients with EBV detected in peripheral blood by a polymerase chain reaction (PCR) (6–8). However, no studies comparing the different EBV detection methods, such as *in situ* hybridization (ISH) in tissue sections and PCR assays using blood specimens, have been conducted on patients with PTC-NOS nor AITL.

This study aimed to investigate the impact of EBV status on the prognosis of patients with PTCL and to compare the use of the two methods for detecting EBV: EBV-encoded small RNA *in situ* hybridization and PCR of DNA polymerase gene. We also assessed the role of hematopoietic stem cell transplantation (HSCT) in these patients.

## Materials and Methods

### Study Population

The study protocol was approved by the Institutional Review Board and Ethics Committee of the Catholic Medical Center in South Korea (KC21RISI0603). The need for informed consent was waived due to the retrospective nature of the study.

This study included patients diagnosed with AITL or PTCL-NOS between May 2009 and May 2019 at the Catholic Hematology Hospital in South Korea. The inclusion criteria were patients with biopsy-proven lymphoma and EBV status analyzed using both ISH and PCR. Patients either without PCR or ISH testing were excluded from the study. All cases were confirmed as PTCL-NOS or AITL using the morphological and immunophenotypic diagnostic criteria of the World Health Organization (3). Patients were evaluated before treatment in terms of performance status, physical examination, complete and differential blood counts, blood chemistry, lactate dehydrogenase levels, chest radiography, and bone marrow biopsy. Computed tomography (CT) of the chest, abdomen, and pelvis and positron emission tomography (PET) were used to determine cancer stage in the cohort.

### Treatment and Response

ProMACE-CytaBOM, CHOP, or SMILE chemotherapy regimens were used, as mentioned in previous studies. The ProMACE-CytaBOM regimen consisted of doxorubicin, cyclophosphamide, and etoposide on day 1 followed by bleomycin, cytosine arabinoside, and vincristine on day 8 as well as prednisone on days 1 – 14. The CHOP regimen consisted of doxorubicin, cyclophosphamide, and vincristine on day 1 and prednisone on days 1 – 5 and was repeated every 21 days (9). The SMILE regimen consisted of methotrexate on day 1, ifosfamide and etoposide on days 2 – 4, and intravenous L-asparaginase based on the existing protocol (10). The doses were adjusted for all patients based on their performance status in addition to hematological, neurological, and infusion-related adverse effects.

Response evaluation was done quarterly for all patients during the first year, biannually for the next three years, and annually thereafter to detect disease recurrence. The response criteria for malignant lymphoma included complete remission (CR; no evidence of disease), partial remission (PR; disease regression but with a measurable remnant and no new lesions), progressive disease (PD; new lesion ≥ 50% of the size of the previous lesion), and stable disease (SD; absence of CR, PR, and PD. Progress free survival (PFS) is defined as the time from diagnosis until lymphoma progression or death as a result of any cause. Overall survival(OS) is defined as the time from diagnosis until death as a result of any cause (11). The cumulative incidence of relapse (CIR) represented the time between disease diagnosis and relapse.

All patients with AITL and PTCL who achieved CR were initially recommended for frontline autologous HSCT. Our department recommended salvage allogeneic HSCT in refractory or relapsed after CR and PR. For conditioning regimen for autologous HSCT, dose reduction strategy of a BU-MEL-TT regimen were used as previous described (12). BU-MEL-TT regimen consist of 2.4 mg/kg busulfan for three consecutive days (total 7.2 mg/kg), 40 mg/m^2^ melphalan for 2 days (total 80 mg/m^2^), and 200 mg/m^2^/thiotepa for two consecutive days (total 400 mg/m^2^). The standard regimen for allogeneic HSCT, RIC FMT regimen previously published (13), consisted of 30 mg/m^2^ fludarabine for 6 consecutive days (total 180 mg/m^2^) and 70 mg/m^2^ melphalan for 1 day with TBI of 800 cGy in 4 fractionated doses for 2 days. Graft versus host disease prophylaxis mainly consisted of cyclosporine for all sibling transplants or tacrolimus for unrelated or haploidentical transplants) with methotrexate (5 mg/m2 for tacrolimus and 10 mg/m2 for cyclosporine on days after transplant 1, 3, 6, and 11).

### Real-Time PCR for Plasma EBV

Blood samples were collected in tubes containing ethylenediaminetetraacetic acid, and DNA was extracted using the Real-Q EBV quantification kit (BioSewoom Inc., Seoul, Korea). Real-time quantitative PCR assays were performed for EBV-specific sequences using the ABI Prism 7500 system (Applied Biosystems Inc., Foster City, CA, USA). Copy numbers were analyzed using a standard curve. The lower threshold for test sensitivity was 5 × 10^2^ copies/mL, and patients with lower values were considered EBV-DNA-negative.

### Tissue ISH

The presence of EBV-specific small RNAs was investigated by ISH. EBV-encoded small RNA (EBER) oligonucleotides were added to formalin-fixed paraffin-embedded sections in accordance with the Inform EBER Probe Assay Protocol (Ventana Medical Systems Inc., Oro Valley, AZ, USA).

### Statistical Analysis

All categorical variables were analyzed using the chi-squared or Fisher’s exact tests whereas the Mann-Whitney u test and Student’s t-test were used for continuous ones.

PFS, OS, and CIR were assessed using Kaplan-Meier curves and compared using the log-rank test. In order to identify the factors that significantly affected survival in the univariate analysis, a Cox proportional-hazards model was used. All predictors with a p-value of <.1 were included in the multivariate analysis. The reported p-values were two-sided, and statistical significance was set at p <.05. All statistical analyses were performed using the R software (version 3.4.3).

## Results

### Patient Characteristics

In total, 140 patients with AITL (n = 68) or PTCL-NOS (n = 72) were included in this study. We defined the EBV-positive group as either detected by PCR or ISH, which was comprised 105 patients. Neither detected by PCR and ISH was 35 patients with EBV-negative group. The median age was 55 years (range, 18–83 years) in the EBV-positive group and 55 years (range, 27–77 years) in the EBV-negative group. Using the International Prognostic Index (IPI), 45.7% of patients in the EBV-positive group scored 0–2, and 54.3% scored 3–5. In the EBV-negative group, these percentages were 48.6% and 51.4%, respectively. There were more EBV-positive cases among patients with AITL (57.1%, n = 60) than among patients with PTCL-NOS (42.9%, n =45). Patient characteristics are summarized in **Table 1**.

**Table 1:** Characteristics of patients with PTCL-NOS or AITL.

Characteristic	EBV-negative (n = 35)	EBV-positive (n = 105)	p-value
**Age**			.465
Median (years)	55 (27–77)	55 (18-83)	
Age ≤ 60, n (%)	26 (74.3)	69 (65.7)	
Age > 60, n (%)	9 (25.7)	36 (34.3)	
**Sex**			.999
Female, n (%)	16 (45.7)	46 (43.8)	
Male, n (%)	19 (54.3)	59 (56.2)	
**Histological subtype**			.001
AITL, n (%)	8 (22.9)	60 (57.1)	
PTCL-NOS, n (%)	27 (77.1)	45 (45.9)	
**Poor performance status**			.347
ECOG 0 – 1, n (%)	21 (60)	74 (70.5)	
ECOG > 1, n (%)	14 (40)	31 (29.5)	
**Extranodal involvement ≥ 2**			.238
n (%)	12/35 (34.3)	50/105 (47.6)	
**Bone marrow involvement**			.764
n (%)	15/35 (42.9)	40/105 (38.1)	
**Ann Arbor stage**			.999
I – II, n (%)	8 (22.9)	25 (23.8)	
III – IV, n (%)	27 (77.1)	80 (76.2)	
**High serum LDH**			.919
n (%)	22/35 (62.9)	69/105 (65.7)	
**High serum β-microglobulin**			.494
n (%)	15/35 (50.0)	43/105 (59.7)	
**CD4 positive**			.565
n (%)	25/35 (71.4)	82/105 (78.1)	
**CD8 positive**			.470
n (%)	14/35 (40.0)	33/105 (31.4)	
**CD30 positive**			.038
n (%)	9/30 (30.0)	41/75 (54.7)	
**Ki-67 ≥ 80**			.245
n (%)	14/35 (40.0)	29/105 (27.6)	
**IPI score**			.922
0 – 2 Low/low-intermediate, n (%)	17 (48.6)	48 (45.7)	
3 – 5 High-intermediate/high, n (%)	18 (51.4)	57 (54.3)	
**PIT score**			.735
1 – 2 Low/low-intermediate, n (%)	25 (71.4)	80 (76.2)	
3 – 4 High-intermediate/high, n (%)	10 (28.4)	25 (23.8)	
**mPIT score**			.437
1– 2 Low/intermediate, n (%)	27 (77.1)	89 (84.8)	
3 High, n (%)	8 (22.9)	16 (15.2)	

AITL, angioimmunoblastic T-cell lymphoma; ECOG, Eastern Cooperative Oncology Group; IPI, International Prognostic Index; LDH, lactate dehydrogenase; PIT, Prognostic Index for T-cell lymphoma; mPIT, modified Prognostic Index for T-cell lymphoma; PTCL-NOS, peripheral T-cell lymphoma not otherwise specified.

### Treatment Outcomes

Chemotherapy was administered to all patients, 75 (53.6%) of whom had an IPI score of 3–5. ProMACE-CytaBOM (n = 52), CHOP (n = 73), and SMILE (n = 15) regimens were administered to the patients. Response evaluation was performed after three or four cycles of chemotherapy. CR was found in 81 patients (57.9%), and PR, PD or SD were observed in 59 patients (42.1%). Among the 55 patients with EBV-positive PTCL who achieved CR with the first treatment, 29 relapsed (52.7%). In comparison, among the 26 patients with CR in EBV-negative group, 11 relapsed (42.3%). Salvage chemotherapy resulted in CR in 14 patients with EBV-positive PTCL who failed to achieve CR in the first chemotherapy (28.0%) and in three patients with EBV-negative PTCL (33.3%).

In the subgroup analysis, significant differences were observed in the CR rates between patients with positive or negative PCR results (hazard ratio [HR] = 0.65, 95% confidence interval [CI]: 0.44 – 0.97, p = .04). Among patients with negative PCR results, those who were ISH-positive for EBV also relapsed more frequently compared to patients with negative ISH results (HR = 2.17, 95% CI: 1.03 – 4.56, p = .035) (**Supplementary Figure 1**).

**Figure 1:**
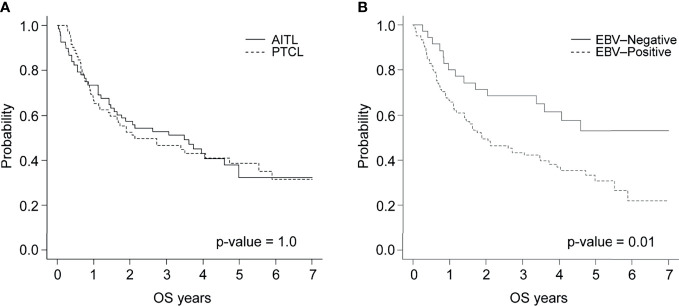
Overall survival by **(A)** tumor subtype and **(B)** EBV status.

After a median follow-up of 33 months (ranging from 0.5 – 123 months), the survival rates of patients with AITL or PTCL-NOS were 52.9% and 46.6%, respectively, with no statistically significant difference between these groups (p = 1.0) (**Figure 1A**). The 3-year PFS was significantly lower (p = .02) in the patients with EBV-positive PTCL (29.1%, n = 105) compared to their EBV-negative counterparts (57.1%, n = 35). Patients in the EBV-positive group were less likely to survive compared to those in the EBV-negative group (43.3% and 68.6%, respectively, p = .01) (**Figure 1B**).

The univariate analysis identified factors associated with increased mortality, which included advanced age (>60 years), high serum β-microglobulin, high and high-intermediate IPI, and the prognostic index for T-cell lymphoma scores as well as EBV positivity. In the multivariate analysis, patients with EBV-positive PTCL were more likely to die (HR = 2.07, 95% CI: 1.19 – 3.65, p = .011) as were those with high and high-intermediate IPI scores (HR = 1.71, 95% CI: 1.11 – 2.66, p = .016).

### Distribution and Survival of EBV-Positive AITL and PTCL-NOS

**Figure 2:**
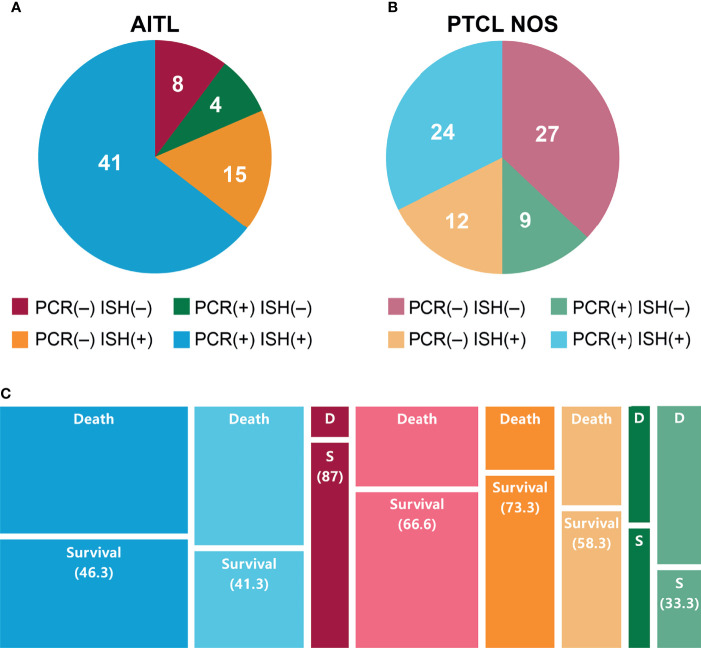
EBV infection in patients with **(A)** AITL or **(B)** PTCL-NOS according to PCR and ISH. **(C)** Survival of patients with AITL or PTCL-NOS after 2 years based on EBV PCR and ISH status.

In the AITL group, 41 patients (60.3%) were both PCR- and ISH-positive, 15 (22.1%) were only ISH-positive, 4 (5.9%) were only PCR-positive, and 8 (11.8%) were negative for both (**Figure 2A**). In the PTCL-NOS group, 24 patients (33.3%) were both PCR- and ISH-positive, 12 (16.7%) were only ISH-positive, 9 (12.5%) were only PCR-positive, and 27 (37.5%) were negative for both (**Figure 2B**).

Among patients with both PCR and ISH positivity, the 2-year survival rates were 46.3% and 41.3% for those with AITL and PTCL-NOS, respectively. These percentages were 87.5% and 66.6% in the PCR- and ISH-negative group, 73.3% and 58.3% in the ISH-only positive group, and 50.0% and 33.3% in the PCR-only positive group (**Figure 2C** and **Supplementary Figure 2A**).

### Subgroup Survival Analysis Among Patients With EBV-Positive and EBV-Negative PTCL With or Without Treatment by HSCT

**Figure 3:**
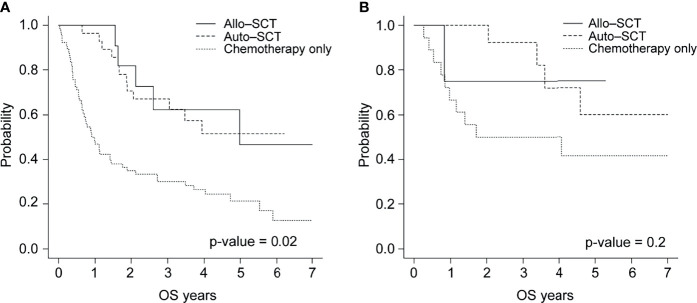
Overall survival of patients with **(A)** EBV-positive or **(B)** EBV-negative PTCL by treatment type (allogeneic HSCT, autologous HSCT, and chemotherapy).

In the EBV-positive group, the 3-year OS rate was 67.0% for the 28 patients treated with autologous HSCT, 62.3% for the 11 treated with allogeneic HSCT, and 30.2% for the 66 treated with chemotherapy only (p <.02). However, no statistically significant differences were observed in the OS of the EBV-negative group (**Figure 3**).

## Discussion

Although there have been several studies on the prognosis of EBV-positive T-cell lymphomas (14), there are few studies that compare the prognosis patients with AITL or PTCL-NOS. EBV infection is rarer in the West than in Asia, and the proportion of T- and NK-cell lymphomas is higher in East Asia than in other regions (15, 16). Because differences in racial and regional genetic factors could influence the immune response to EBV (17), evaluating the prognostic impact of EBV infection is essential in Korea.

Our study was based on the similarity in survival outcomes between patients with AITL or PTCL-NOS. Warnke et al. described the challenges of distinguishing AITL from PTCL-NOS due to their morphological and genetic similarities. Furthermore, their study demonstrated that patients with PTCL-NOS who had a genetic signature similar to AITL were prone to poor outcomes (18).

We hypothesized that EBV status could comparably impact AITL and PTCL-NOS. EBV infection is generally evaluated by two methods, and EBV-ISH is currently used to differentiate lymphoma subtypes and gives clues to distinguish AITL from PTCL-NOS (19, 20). Detection of EBV by PCR is used for prognosis prediction (21) and the early detection (22) of various type of cancers. Therefore, we tried to assess this in our cohort.

In our study, we observed a poor prognosis of patients with EBV-positive PTCL detected by ISH or PCR. These two detection methods play different roles in predicting poor outcomes. Blood PCR-positive cases showed resistance to the first-line chemotherapy. In the PCR-negative group, patients with ISH-positive PTCL had a higher recurrence rate compared to their ISH-negative counterparts regardless of a good response to chemotherapy; hence, they were all included in the EBV-positive group. EBV PCR positivity was associated with chemotherapy resistance, which is consistent with published (23). Although a few studies support this trend in patients with PTCL-NOS or AITL, these studies only showed that cases of EBV status detected by PCR and not ISH were associated with poor survival outcomes. However, these studies include cases other types of T-cell lymphoma regardless of different survival outcomes associated with each subtypes, which could obscure the influence of EBV infection (6, 7).

Even though EBV ISH positivity was associated with early relapse in patients with B-cell lymphoma (24, 25) there is a lack of data on TCL. Some studies shown that ISH-positive cases are associated with higher susceptibility of relapse (26) but this has been contradicted by other findings (27). This discordance arises because EBV-ISH and EBV-PCR results should be interpreted together. For example, in the cases of ISH-negative and PCR-positive TCL, which are associated with a poor outcome, the source of the EBV infection could have come from B cells and may need additional treatment such as rituximab administration.

Due to the overlapping characteristics of PTCL-NOS and AITL, similar treatment strategies consisting of autologous HSCT following chemotherapy (28, 29) and salvage allogeneic HSCT have been used in the clinic (30). Despite a few prospective studies that have demonstrated its benefits (31), the role of first-line autologous HSCT is not confirmed (32). However, EBV-associated lymphoma may be amenable to first-line autologous HSCT in TCL. EBV-induced lymphomas have high proliferative signaling and cell death resistance (33). EBV latency in tumor cells increases intracellular ROS activity and the MDR gene expression by activating the STAT1 signaling pathway and phosphorylated STAT1, promoting P-gp expression. P-gp-induced drug efflux resulted in reduced chemo sensitivity (34). Recently, front-line autologous HSCT has been attempted for extranodal NK/T-cell lymphoma (ENKTCL) and has proven useful (35).

As additional treatments, Epstein-Barr nuclear antigen and EBV latent membrane protein-specific cytotoxic T-lymphocytes (CTLs) might be beneficial. CTLs have been used to treat EBV-positive Hodgkin, non-Hodgkin lymphoma and ENKTCL with favorable outcomes (36–38). However, the efficacy of CTLs may be limited in the tumor microenvironment, which impairs the recognition of lymphoma cells by CTLs. For example, the interaction between PD-1 and PD-L1 downregulates T-cell function and leads to CTL exhaustion. As EBV infection systemically affects the immune system, EBV-positive PTCL-NOS and AITL could be candidates for the use of immune checkpoint inhibitors (39–41). Additional studies focusing on different treatment modalities for EBV-positive PTCL-NOS and AITL are warranted.

In observational studies, there is a potential for bias based on the inclusion criteria. Since we excluded the cases where EBV-related tests were omitted, the EBV distribution may be relatively different from the previously published. This explains the relatively higher EBV status on PTCL NOS in our cohort. We did not perform first-line autologous HSCT as in prospective studies (31). Clinicians recommend autologous HSCT to all patients with susceptible as we described, and some cases were not proceeded due to poor physical status or personal issues. Therefore, interpretation of the effect of first-line autologous HSCT and salvage allogeneic HSCT is limited.

In conclusion, testing for EBV status using both PCR and ISH in patients with AITL or PTCL-NOS may be useful, and HSCT may help improve survival rates in of infected patients. A large-scale study of the effect of EBV infection on the pathological mechanisms of PTCL-NOS and AITL is needed, and prospective randomized clinical trials are required to determine the standard treatments for EBV-associated AITL and PTCL-NOS.

## Data Availability Statement

The raw data supporting the conclusions of this article will be made available by the authors, without undue reservation.

## Ethics Statement

The study protocol was approved by the Institutional Review Board and Ethics Committee of the Catholic Medical Center in South Korea (KC21RISI0603). Written informed consent for participation was not required for this study in accordance with the national legislation and the institutional requirements.

## Author Contributions

T-YK performed the research, collected and analyzed the data, in addition to writing the manuscript. G-JM, S-HS, S-AY, J-HY, B-SC, and H-JK provided patients and materials and reviewed the manuscript. S-SP, S-EL, K-SE, and C-KM provided the materials and reviewed the manuscript. Y-JK, SL, and J-WL reviewed the manuscript and analyzed the data. S-GC designed and conducted the study, provided patients and materials, analyzed data, and wrote the manuscript. All authors read and approved the final manuscript.

## Conflict of Interest

The authors declare that the research was conducted in the absence of any commercial or financial relationships that could be construed as a potential conflict of interest.

## Publisher’s Note

All claims expressed in this article are solely those of the authors and do not necessarily represent those of their affiliated organizations, or those of the publisher, the editors and the reviewers. Any product that may be evaluated in this article, or claim that may be made by its manufacturer, is not guaranteed or endorsed by the publisher.

## References

[1] Fiore D Cappelli LV Broccoli A Zinzani PL Chan WC Inghirami G. Peripheral T Cell Lymphomas: From the Bench to the Clinic. Nat Rev Cancer (2020) 20:323–42. doi: 10.1038/s41568-020-0247-0 32249838

[2] Yoon SE Song Y Kim SJ Yoon DH Chen TY Koh Y. Comprehensive Analysis of Peripheral T-Cell and Natural Killer/T-Cell Lymphoma in Asian Patients: A Multinational, Multicenter, Prospective Registry Study in Asia. Lancet Reg Health West Pac (2021) 10:100126. doi: 10.1016/j.lanwpc.2021.100126 34327343 PMC8315366

[3] Swerdlow SH Campo E Pileri SA Harris NL Stein H Siebert R. The 2016 Revision of the World Health Organization Classification of Lymphoid Neoplasms. Blood (2016) 127:2375–90. doi: 10.1182/blood-2016-01-643569 PMC4874220 26980727

[4] Hsi ED Horwitz SM Carson KR Pinter-Brown LC Rosen ST Pro B. Analysis of Peripheral T-Cell Lymphoma Diagnostic Workup in the United States. Clin Lymphoma Myeloma Leuk (2017) 17:193–200. doi: 10.1016/j.clml.2016.10.001 28209473

[5] Dobay MP Lemonnier F Missiaglia E Bastard C Vallois D Jais J-P. Integrative Clinicopathological and Molecular Analyses of Angioimmunoblastic T-Cell Lymphoma and Other Nodal Lymphomas of Follicular Helper T-Cell Origin. Haematologica (2017) 102:e148–51. doi: 10.3324/haematol.2016.158428 PMC5395128 28082343

[6] Kim YR Kim SJ Cheong JW Chung H Eun Jang J Kim Y. Pretreatment Epstein–Barr Virus DNA in Whole Blood Is a Prognostic Marker in Peripheral T-Cell Lymphoma. Oncotarget (2017) 8:92312–23. doi: 10.18632/oncotarget.21251 PMC5696183 29190917

[7] Liang JH Lu L Zhu HY Li W Fan L Li JY. The Prognostic Role of Circulating Epstein–Barr Virus DNA Copy Number in Angioimmunoblastic T-Cell Lymphoma Treated With Dose-Adjusted EPOCH. Cancer Res Treat (2019) 51:150–7. doi: 10.4143/crt.2017.476 PMC6334003 29621877

[8] Haverkos BM Huang Y Gru A Pancholi P Freud AG Mishra A. Frequency and Clinical Correlates of Elevated Plasma Epstein–Barr Virus DNA at Diagnosis in Peripheral T-Cell Lymphomas. Int J Cancer (2017) 140:1899–906. doi: 10.1002/ijc.30566 PMC5323329 27943278

[9] Montserrat E García Conde J Viñolas N López Guillermo A Hernández-Nieto L Zubizarreta A. CHOP vs. ProMACE-CytaBOM in the Treatment of Aggressive Non-Hodgkin’s Lymphomas: Long-Term Results of a Multicenter Randomized Trial. (PETHEMA: Spanish Cooperative Group for the Study of Hematological Malignancies Treatment, Spanish Society of Hematology). Eur J Haematol (1996) 57:377–83. doi: 10.1111/j.1600-0609.1996.tb01396.x 9003479

[10] Yamaguchi M Suzuki R Kwong YL Kim WS Hasegawa Y Izutsu K. Phase I Study of Dexamethasone, Methotrexate, Ifosfamide, L-Asparaginase, and Etoposide (SMILE) Chemotherapy for Advanced-Stage, Relapsed or Refractory Extranodal Natural Killer (NK)/T-Cell Lymphoma and Leukemia. Cancer Sci (2008) 99:1016–20. doi: 10.1111/j.1349-7006.2008.00768.x PMC11158592 18294294

[11] Cheson BD Pfistner B Juweid ME Gascoyne RD Specht L Horning SJ. Revised Response Criteria for Malignant Lymphoma. J Clin Oncol (2007) 25:579–86. doi: 10.1200/JCO.2006.09.2403 17242396

[12] Yoon JH Min GJ Park SS Jeon YW Lee SE Cho BS. Autologous Hematopoietic Cell Transplantation Using Dose-Reduced Intravenous Busulfan, Melphalan, and Thiotepa for High-Risk or Relapsed Lymphomas. Bone Marrow Transplant (2019) 54:330–3. doi: 10.1038/s41409-018-0289-z 30082849

[13] Jeon YW Yoon S Min GJ Park SS Park S Yoon JH. Clinical Outcomes of Fludarabine and Melphalan With an 800 cGy Total Body Irradiation Conditioning Regimen in Patients With Refractory or Relapsed Aggressive Non-Hodgkin Lymphoma Undergoing Allogeneic Hematopoietic Stem Cell Transplantation. Clin Lymphoma Myeloma Leuk (2019) 19:345–355.e7. doi: 10.1016/j.clml.2019.03.023 31014757

[14] Harabuchi Y Takahara M Kishibe K Nagato T Kumai T. Extranodal Natural Killer/T-Cell Lymphoma, Nasal Type: Basic Science and Clinical Progress. Front Pediatr (2019) 7:141. doi: 10.3389/fped.2019.00141 31041299 PMC6476925

[15] Park S Ko YH. Peripheral T Cell Lymphoma in Asia. Int J Hematol (2014) 99:227–39. doi: 10.1007/s12185-014-1520-3 24481942

[16] Cho EY Kim KH Kim WS Yoo KH Koo HH Ko YH. The Spectrum of Epstein–Barr Virus-Associated Lymphoproliferative Disease in Korea: Incidence of Disease Entities by Age Groups. J Korean Med Sci (2008) 23:185–92. doi: 10.3346/jkms.2008.23.2.185 PMC2526432 18436998

[17] Ko YH. EBV-Associated Lymphoproliferative Disorders. Clin Pediatr Hematol Oncol (2021) 28:14–27. doi: 10.15264/cpho.2021.28.1.14

[18] Iqbal J Wright G Wang C Rosenwald A Gascoyne RD Weisenburger DD. Gene Expression Signatures Delineate Biological and Prognostic Subgroups in Peripheral T-Cell Lymphoma. Blood (2014) 123:2915–23. doi: 10.1182/blood-2013-11-536359 PMC4014836 24632715

[19] Nakatsuka S-i Homma K Aozasa K. When to Use *In Situ* Hybridization for the Detection of Epstein–Barr Virus: A Review of Epstein–Barr Virus-Associated Lymphomas. J Hematopathol (2015) 8:61–70. doi: 10.1007/s12308-014-0230-3

[20] Attygalle AD Chuang SS Diss TC Du MQ Isaacson PG Dogan A. Distinguishing Angioimmunoblastic T-Cell Lymphoma From Peripheral T-Cell Lymphoma, Unspecified, Using Morphology, Immunophenotype and Molecular Genetics. Histopathology (2007) 50:498–508. doi: 10.1111/j.1365-2559.2007.02632.x 17448026

[21] Kanakry JA Li H Gellert LL Lemas MV Hsieh WS Hong F. Plasma Epstein–Barr Virus DNA Predicts Outcome in Advanced Hodgkin Lymphoma: Correlative Analysis From a Large North American Cooperative Group Trial. Blood (2013) 121:3547–53. doi: 10.1182/blood-2012-09-454694 PMC3643756 23386127

[22] Chan KCA Woo JKS King A Zee BCY Lam WKJ Chan SL. Analysis of Plasma Epstein–Barr Virus DNA to Screen for Nasopharyngeal Cancer. N Engl J Med (2017) 377:513–22. doi: 10.1056/NEJMoa1701717 28792880

[23] Wang L Wang H Wang JH Xia ZJ Lu Y Huang HQ. Post-Treatment Plasma EBV-DNA Positivity Predicts Early Relapse and Poor Prognosis for Patients With Extranodal NK/T Cell Lymphoma in the Era of Asparaginase. Oncotarget (2015) 6:30317–26. doi: 10.18632/oncotarget.4505 PMC4745801 26210287

[24] Park S Lee J Ko YH Han A Jun HJ Lee SC. The Impact of Epstein–Barr Virus Status on Clinical Outcome in Diffuse Large B-Cell Lymphoma. Blood (2007) 110:972–8. doi: 10.1182/blood-2007-01-067769 17400912

[25] Crombie JL LaCasce AS. Epstein Barr Virus Associated B-Cell Lymphomas and Iatrogenic Lymphoproliferative Disorders. Front Oncol (2019) 9:109. doi: 10.3389/fonc.2019.00109 30899698 PMC6416204

[26] Dupuis J Emile JF Mounier N Gisselbrecht C Martin-Garcia N Petrella T. Prognostic Significance of Epstein–Barr Virus in Nodal Peripheral T-Cell Lymphoma, Unspecified: A Groupe D’Etude Des Lymphomes De L’adulte (GELA) Study. Blood (2006) 108:4163–9. doi: 10.1182/blood-2006-04-017632 16902151

[27] Eladl AE Shimada K Suzuki Y Takahara T Kato S Kohno K. Status has Prognostic Implication Among Young Patients With Angioimmunoblastic T-Cell Lymphoma. Cancer Med (2020) 9:678–88. doi: 10.1002/cam4.2742 PMC6970042 31793218

[28] Moskowitz AJ. Practical Treatment Approach for Angioimmunoblastic T-Cell Lymphoma. J Oncol Pract (2019) 15:137–43. doi: 10.1200/JOP.18.00511 PMC7850668 30861367

[29] Tang T Khoo LP Lim C Ham JS Kim SJ Hong H. Outcomes of Patients With Peripheral T-Cell Lymphoma in First Complete Remission: Data From Three Tertiary Asian Cancer Centers. Blood Cancer J (2017) 7:653. doi: 10.1038/s41408-017-0030-y 29242582 PMC5802553

[30] Goldberg JD Chou JF Horwitz S Teruya-Feldstein J Barker JN Boulad F. Long-Term Survival in Patients With Peripheral T-Cell Non-Hodgkin Lymphomas After Allogeneic Hematopoietic Stem Cell Transplant. Leuk Lymphoma (2012) 53:1124–9. doi: 10.3109/10428194.2011.645818 PMC4054938 22136377

[31] d’Amore F Relander T Lauritzsen GF Jantunen E Hagberg H Anderson H. Up-Front Autologous Stem-Cell Transplantation in Peripheral T-Cell Lymphoma: NLG-T-01. J Clin Oncol (2012) 30:3093–9. doi: 10.1200/JCO.2011.40.2719 22851556

[32] Fossard G Broussais F Coelho I Bailly S Nicolas-Virelizier E Toussaint E. Role of Up-Front Autologous Stem-Cell Transplantation in Peripheral T-Cell Lymphoma for Patients in Response After Induction: An Analysis of Patients From LYSA Centers. Ann Oncol (2018) 29:715–23. doi: 10.1093/annonc/mdx787 29253087

[33] Mesri EA Feitelson MA Munger K. Human Viral Oncogenesis: A Cancer Hallmarks Analysis. Cell Host Microbe (2014) 15:266–82. doi: 10.1016/j.chom.2014.02.011 PMC3992243 24629334

[34] Nam YS Im KI Kim N Song Y Lee JS Jeon YW. Down-Regulation of Intracellular Reactive Oxygen Species Attenuates P-Glycoprotein-Associated Chemoresistance in Epstein–Barr Virus-Positive NK/T-Cell Lymphoma. Am J Transl Res (2019) 11:1359–73. PMC6456522 30972167

[35] Yhim HY Kim JS Mun YC Moon JH Chae YS Park Y. Clinical Outcomes and Prognostic Factors of Up-Front Autologous Stem Cell Transplantation in Patients With Extranodal Natural Killer/T Cell Lymphoma. Biol Blood Marrow Transplant (2015) 21:1597–604. doi: 10.1016/j.bbmt.2015.05.003 25963920

[36] Cho SG Kim N Sohn HJ Lee SK Oh ST Lee HJ. Long-Term Outcome of Extranodal NK/T Cell Lymphoma Patients Treated With Postremission Therapy Using EBV LMP1 and LMP2a-Specific CTLs. Mol Ther (2015) 23:1401–9. doi: 10.1038/mt.2015.91 PMC4817864 26017177

[37] Bollard CM Gottschalk S Torrano V Diouf O Ku S Hazrat Y. Sustained Complete Responses in Patients With Lymphoma Receiving Autologous Cytotoxic T Lymphocytes Targeting Epstein–Barr Virus Latent Membrane Proteins. J Clin Oncol (2014) 32:798–808. doi: 10.1200/JCO.2013.51.5304 24344220 PMC3940538

[38] Icheva V Kayser S Wolff D Tuve S Kyzirakos C Bethge W. Adoptive Transfer of Epstein–Barr Virus (EBV) Nuclear Antigen 1–Specific T Cells as Treatment for EBV Reactivation and Lymphoproliferative Disorders After Allogeneic Stem-Cell Transplantation. J Clin Oncol (2013) 31:39–48. doi: 10.1200/JCO.2011.39.8495 23169501

[39] Bi XW Wang H Zhang WW Wang JH Liu WJ Xia ZJ. PD-L1 Is Upregulated by EBV-Driven LMP1 Through NF-κb Pathway and Correlates With Poor Prognosis in Natural Killer/T-Cell Lymphoma. J Hematol Oncol (2016) 9:109. doi: 10.1186/s13045-016-0341-7 27737703 PMC5064887

[40] Nagato T Ohkuri T Ohara K Hirata Y Kishibe K Komabayashi Y. Programmed Death-Ligand 1 and Its Soluble Form Are Highly Expressed in Nasal Natural Killer/T-Cell Lymphoma: A Potential Rationale for Immunotherapy. Cancer Immunol Immunother (2017) 66:877–90. doi: 10.1007/s00262-017-1987-x PMC11028583 28349165

[41] Kwong YL Chan TSY Tan D Kim SJ Poon LM Mow B. PD1 Blockade With Pembrolizumab is Highly Effective in Relapsed or Refractory NK/T-Cell Lymphoma Failing L-Asparaginase. Blood (2017) 129:2437–42. doi: 10.1182/blood-2016-12-756841 28188133

