# Supplementary material for: Impact of Epstein-Barr Virus on Peripheral T-Cell Lymphoma Not Otherwise Specified and Angioimmunoblastic T-Cell Lymphoma
Source: Front Oncol. 2022 Jan 11;11:797028. doi: 10.3389/fonc.2021.797028 (PMC8786732; doi:10.3389/fonc.2021.797028)
Supplement: Supplementary file 1 [file DataSheet_1.docx]

Supplementary Material

# Supplementary Figures

**
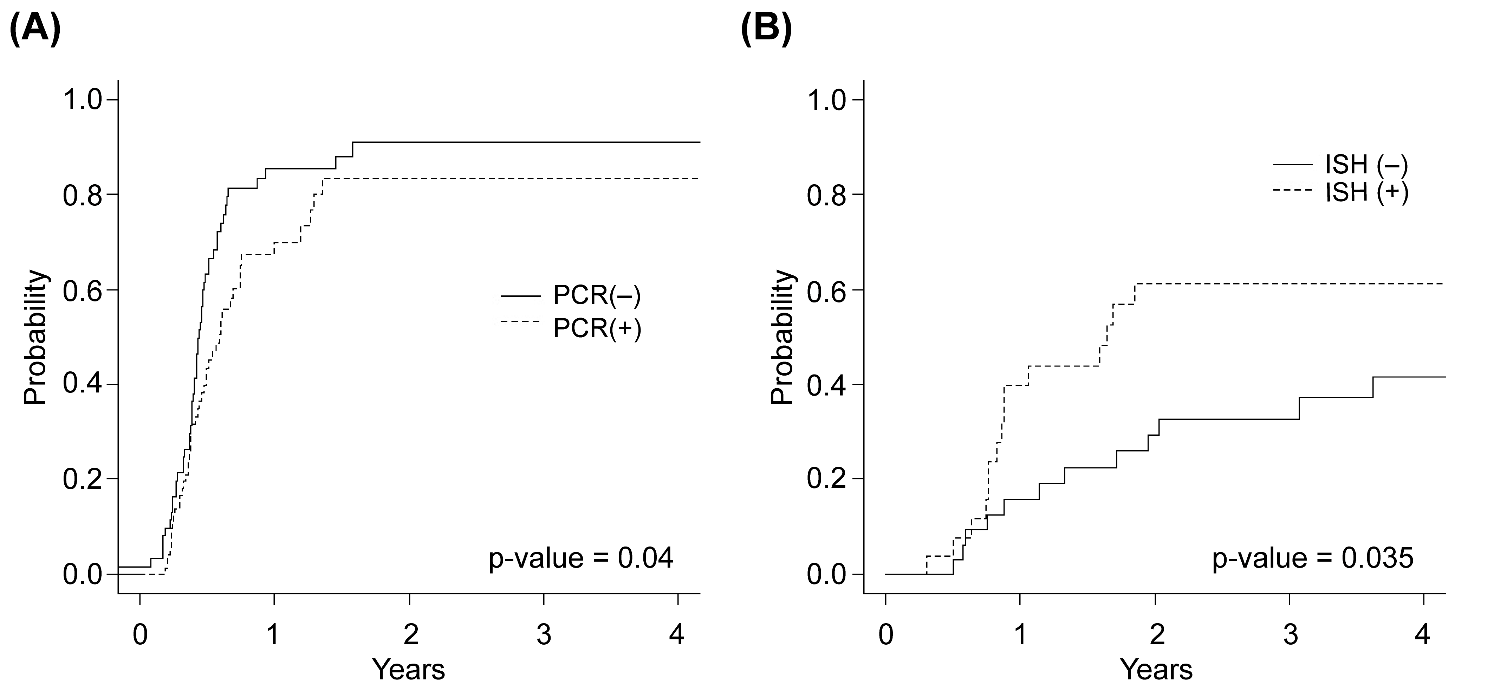
**

**Supplementary Figure 1.** **(A)** Complete remission rates by PCR-positivity **(B)** Cumulative incidence rate of relapse in patients with PCR-negative EBV stratified by ISH positivity.

EBV, Epstein-Barr virus; ISH, *in situ* hybridization; PCR, polymerase chain reaction


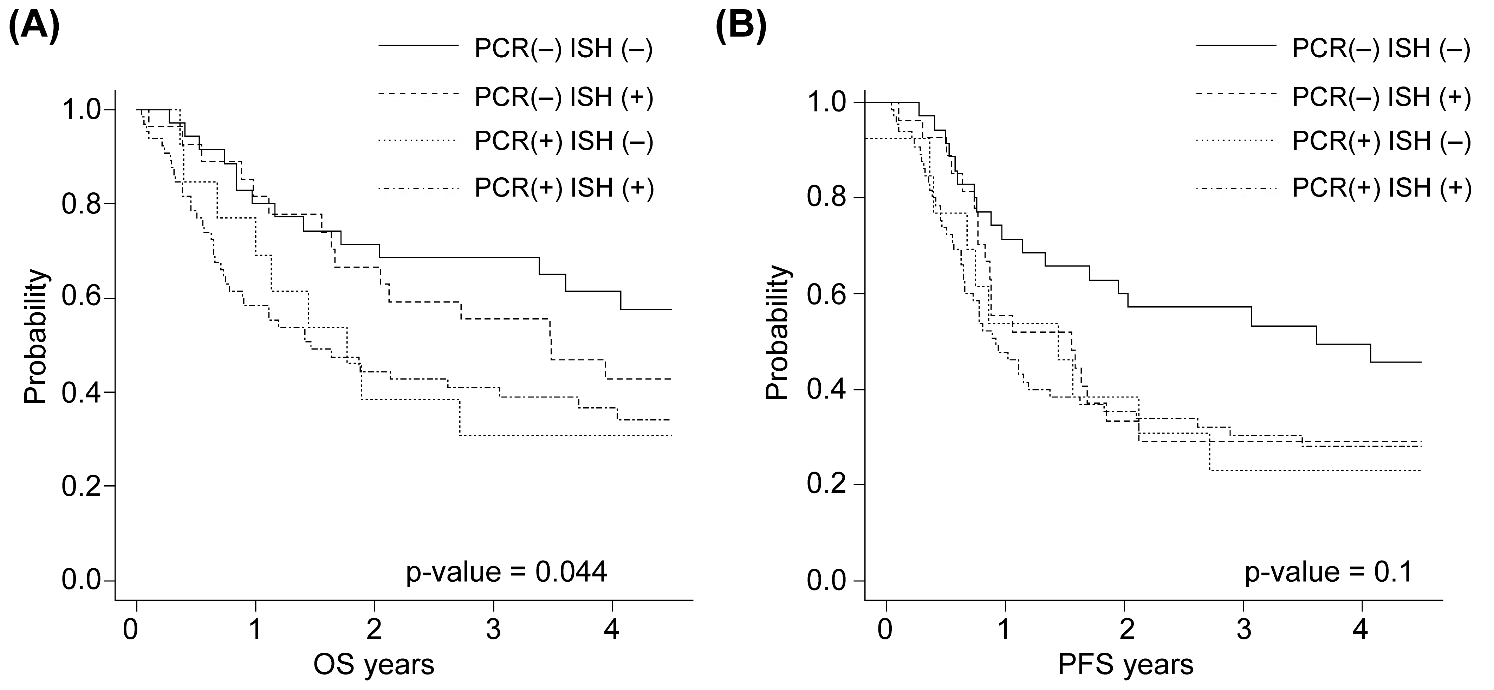


**Supplementary Figure 2.** **(A)** Overall survival of all patients **(B)** Progression-free survival of EBV-negative patients stratified by testing (PCR [-] with ISH[-], PCR[-] with ISH [+], PCR [+] with ISH[-], and PCR [+] with ISH [+]).

EBV, Epstein-Barr virus; ISH, *in situ* hybridization; PCR, polymerase chain reaction
